# Butein Ameliorates Oxidative Stress in H9c2 Cardiomyoblasts through Activation of the NRF2 Signaling Pathway

**DOI:** 10.3390/antiox11081430

**Published:** 2022-07-23

**Authors:** Tsendsuren Tungalag, Kye Won Park, Dong Kwon Yang

**Affiliations:** 1 Department of Veterinary Pharmacology and Toxicology, College of Veterinary Medicine, Jeonbuk National University, Iksan 54596, Korea; mgljuuh@jbnu.ac.kr; 2 Department of Food Science and Biotechnology, Food Clinical Research Center, Sungkyunkwan University, Suwon 16419, Korea

**Keywords:** oxidative stress, butein, reactive oxygen species (ROS), ER stress, Nrf2

## Abstract

Oxidative stress, defined as an imbalance between reactive oxygen species (ROS) production and the antioxidant defense system, contributes to the pathogenesis of many heart diseases. Therefore, oxidative stress has been highlighted as a therapeutic target for heart disease treatment. Butein, a tetrahydroxychalcone, has potential biological activities, especially antioxidant properties. However, the effect of butein on oxidative-stressed heart cells has been poorly studied. Thus, we sought to identify the antioxidant effects of butein in H9c2 cardiomyoblasts. To elucidate these antioxidant effects, various concentrations of butein were used to pretreat H9c2 cells prior to H_2_O_2_ treatment. Thereafter, measures of oxidative damages, such as ROS production, antioxidant expression levels, and apoptosis, were evaluated. Butein effectively increased cell viability and rescued the cells from oxidative damage through the inhibition of ROS production, apoptosis, and increased antioxidant expression. Furthermore, butein dramatically inhibited mitochondrial dysfunction and endoplasmic reticulum (ER) stress, which are the main ROS inducers. Nrf2 protein translocated from the cytosol to the nucleus and consequently activated its target genes as oxidative stress suppressors. These findings demonstrate that butein has potential antioxidant effects in H9c2 cardiomyoblasts, suggesting that it could be used as a therapeutic substance for the treatment of cardiac diseases.

## 1. Introduction

Oxidative stress arises from an imbalance between reactive oxygen species (ROS) and the endogenous antioxidant system. ROS are highly reactive molecules derived from oxygen (O_2_) [1]. Indeed, physiological levels of ROS are crucial signaling molecules that act as second messengers in normal physiological processes and have beneficial influences in cell physiology, including wound healing, repair processes, and pathogen defense [2]. However, the accumulation of ROS disrupts the redox homeostasis inside the cell, further leading to oxidative stress. Oxidative stress promotes a wide variety of cellular processes that can provoke cellular dysfunction, such as apoptosis and necrosis. Therefore, a sophisticated antioxidant system exists inside the cell to regulate ROS production, which causes oxidative stress. The antioxidants involved in the antioxidant system can be categorized as enzymatic and non-enzymatic antioxidants based on their biological activities [3]. Enzymatic antioxidants, comprising endogenous enzymes, break down and remove free radicals [3]. Non-enzymatic antioxidants, such as vitamin A, vitamin C, carotenoids, and β-carotenes, have antioxidant effects by promoting anti-oxidative enzymes or by interrupting free radical oxidative chain reactions [3]. The combined action of both enzymatic and non-enzymatic antioxidants prevents the formation of free radicals or repairs cellular damage caused by them [4].

Evidence indicates that oxidative stress is a major cause of the progression of various diseases, including diabetes, cancer, metabolic disorders, and cardiovascular diseases [5]. The role of oxidative stress in the onset of diseases is as both the primary cause and a secondary contributor to disease progression. Notably, if oxidative stress acts as a primary factor, antioxidant therapy often fails to block the progression of disease once tissue damage begins [6]. In particular, the heart is susceptible to oxidative stress. Cardiac cells contain a large number of mitochondria as a main source of ROS production due to the high consumption of O_2_ for energy production [7]. In addition, antioxidants are present at low levels in the heart and are considered another cause of oxidative stress in the heart [8]. Therefore, the accumulation of ROS causes mitochondrial dysfunction and apoptosis in cardiac cells, which further progresses to cardiac disease [9]. Indeed, oxidative stress has been associated with the progression of many cardiac diseases, such as heart failure, myocardial ischemia-reperfusion injury, and cardiac arrythmias [10]. Therefore, antioxidative therapies in which exogenous antioxidants are supplied have been studied for cardiac disease prevention and treatment. Consequently, many antioxidants have been identified, but their effects are limited or controversial.

Butein (3,4,2′,4′-tetrahydroxychalone), a polyphenol, has a variety of biological properties, such as anti-inflammatory [11], anti-cancer [12], and antioxidative [13] activities. In a clinical trial, butein-containing flavonoids markedly decreased tumor size with good tolerability in patients with gastric cancer [14]. In particular, a previous study showed that butein has the inhibitory effects against hydrogen peroxide (H_2_O_2_)-induced hemolysis and lipid peroxidation due to its antioxidative properties in rat erythrocytes [15]. Another study also showed that butein, as an antioxidant, suppressed the activation of hepatic stellate cells by ethanol [16].

In the present study, we demonstrated that butein has an inhibitory effect on H_2_O_2_-induced oxidative stress in H9c2 cardiomyoblasts by inhibiting mitochondrial dysfunction and ER stress through the preservation of Nrf2 protein and its target genes with antioxidant functions.

## 2. Materials and Methods

### 2.1. Chemicals, Reagents, and Kits

Butein was obtained from Sigma-Aldrich (St. Louis, MO, USA). All reagents used for cell culture were purchased at ThermoFisher Scientific (Chelmsford, MA, USA). The reagents used for functional studies were purchased from Sigma, ThermoFisher, and Roche Diagnostics (Basel, Switzerland).

### 2.2. Cell Culture and Treatment with Butein and H_2_O_2_

H9c2 cardiomyoblasts were obtained from the Cell Culture Cluster (Seoul, Korea). The cells were cultured in DMEM with 10% FBS and 1% antibiotics and maintained in a incubator with 5% CO_2_ and 95% air at 37 °C [17]. After dissolving butein in dimethyl sulfoxide (DMSO), various concentrations of butein ranging from 0.5–10 µM were used to treat cells for 24, 48, and 72 h to determine its cytotoxicity. For functional studies, 1, 3, and 5 µM concentrations of butein were used to treat cells for 24 h. Then, 500 µM H_2_O_2_ was treated for oxidative stress for 24 h.

### 2.3. Cell Viability Assay

The cells were plated in 96-well culture plates at a density of 2 × 10^3^ cells. After they were grown until 80% confluency, cells were treated with butein and/or H_2_O_2_. Cell viability was measured using 3-[4,5-dimethylthiazol-2-yl]-2, 5-diphenyltetrazolium bromide (MTT; Sigma) [18]. Briefly, 0.5 mg/mL MTT was added to each well, and the wells were then incubated for 2 h at 37 °C. DMSO was then added to dissolve the formazan crystals. Absorbance was measured at 570 nm using a spectrophotometer (Molecular Devices, Sunnyvale, CA, USA).

### 2.4. Hoechst 33342 Staining

The detection of apoptotic cells was determined by nuclear staining using Hoechst 33342 dye [19]. Briefly, the cells that were treated with butein and/or H_2_O_2_ were fixed with 4% paraformaldehyde (PFA; Sigma) for 30 min at room temperature (RT). Then, 10 µg/mL Hoechst dye (ThermoFisher) was added for 30 min at 37 °C. The nuclei were imaged by a fluorescence microscope (Olympus, Tokyo, Japan).

### 2.5. Analysis of Intracellular ROS Production

To detect intracellular ROS production, DCFH-DA (ThermoFisher) dye was used [20]. Briefly, the cells were seeded in 6-well plates at a density of 1 × 10^5^ cells per well and were then treated with butein and/or H_2_O_2_. After that, 1 µM DCFH-DA dye was treated to the cells for 30 min at 37 °C after washing thrice with PBS. Finally, they were imaged with a fluorescence microscope (Olympus).

### 2.6. MitoSOX Red Staining

The ROS in mitochondria was observed by using MitoSOX Red mitochondrial superoxide indicator (ThermoFisher) [21]. Briefly, after the cells were seeded in 6-well plates, they were treated with butein and/or H_2_O_2_. After that, the cells were added to 5 µM MitoSOX reagent for 10 min at 37 °C. After washing thrice with PBS, they were then imaged by a live-cell imaging system (Oxford Instruments, Oxfordshire, UK).

### 2.7. Immunostaining of Nrf2 Protein

After treatment with butein and/or H_2_O_2_, the cells were fixated with 4% PFA for 10 min at RT. They were then permeabilized with 1% Triton X-100 in PBS for 10 min. After blocking by 5% BSA for 1 h, the cells were incubated with Nrf2 primary antibody (sc-365949, 1:200, Santa Cruz Biotechnology, Texas, TX, USA) overnight at 4 °C followed by incubation with a tetramethylrhodamine (TRITC)-conjugated secondary antibody (1:5000; ThermoFisher) for 1 h at RT. After that, the nuclei were stained with 4,6-diamidino-2-phenyindole (DAPI, Vector Laboratories, inc., Burlingame, CA, USA) and were further imaged by a fluorescence microscope (Olympus).

### 2.8. Western Blot Analysis

The cells that treated with butein and/or H_2_O_2_ were lysed in RIPA buffer (1% NP-40, 50 mM Tris–HCl [pH 7.4], 150 mM NaCl, and 10 mM NaF) supplemented with the protease inhibitors (Roche) and phosphatase inhibitors (Roche Diagnostics). The proteins were separated by SDS-PAGE and transferred onto PVDF membranes (Bio-Rad, Hercules, CA, USA). After blocking with 5% BSA in TBST buffer for 1 h at RT, they were incubated with the appropriate primary antibodies overnight at 4 °C. The membranes were further incubated with HRP-conjugated secondary antibodies (1:5000; AbFrontier, Seoul, Korea). The ECL reagents and a UVITEC system (Cleaver Scientific, Warwickshire, UK) were used to develop and visualize the protein bands. The antibodies are listed in Supplementary Table S1.

### 2.9. Nuclear Fractionation of H9c2 Cardiomyocytes

Nuclear fractionation was performed using a nuclear-extraction reagents kit according to the manufacturer’s instructions (abcam). In brief, the cells were lysed with hypotonic buffer (10 mM HEPES, 1.5 mM MgCl_2_, 10 mM KCl, 0.5 mM DTT, 0.05% NP40, and pH 7.9) supplemented with protease inhibitors followed by incubating for 10 min on ice. The nuclear pellet was harvested by centrifugation at 3000× *g* for 10 min at 4 °C. After the pellet was washed thrice, it was suspended with the lysis buffer (5 mM HEPES, 1.5 mM MgCl_2_, 0.2 mM EDTA, 0.5 mM DTT, 26% glycerol (*v*/*v*), and pH 7.9). After incubating for 30 min on ice, it was collected after centrifugation at 24,000× *g* for 20 min at 4 °C.

### 2.10. Quantitative Real-Time Polymerase Chain Reaction (qRT-PCR)

cDNA was synthesized from 1 µg total RNA using ImProm II RT kit (Promega, Madison, WI, USA) after extracting total RNA by Monarch total RNA extraction kit (Promega). The qPCR kit from Kapa Biosystems (Boston, MA, USA) with a qRT-PCR machine (Takara, Shiga, Japan) was used. GAPDH gene was used for normalization. The primer sequences are shown in Supplementary Table S2.

### 2.11. Statistical Analysis

Values are shown as the SEM. Statistical analysis was performed using one-way ANOVA with a Bonferroni post hoc test using Prism 8.02 (GraphPad, San Diego, CA, USA). *p* values less than 0.05 were considered statistically significant.

## 3. Results

### 3.1. Butein Has No Cytotoxicity on H9c2 Cardiomyoblasts

**Figure 1:**
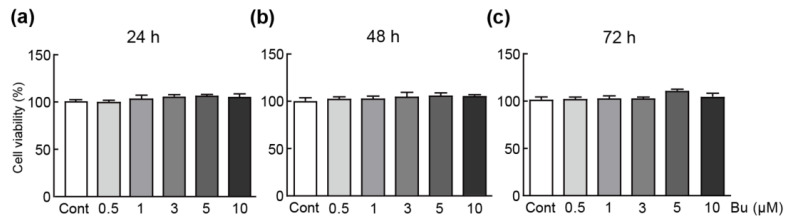
Cytotoxic effects of butein in H9c2 cardiomyoblasts. An MTT assay was performed to measure the cell viability. The cells were treated with various concentrations of butein (0.5, 1, 3, 5, and 10 µM) for 24 (**a**), 48 (**b**), and 72 h (**c**). The results are shown as the mean ± SEM in triplicate. Cont; control, Bu; butein.

To determine whether butein is toxic to cells, they were treated with various concentrations of butein ranging from 0.5 to 10 µM for 24, 48, and 72 h. An MTT assay was performed to determine the viability of cells treated with butein. The results showed that treatment with all concentrations and times of butein did not significantly change the cell viability (Figure 1a–c). Therefore, these results show that butein does not exhibit cytotoxic effects on H9c2 cardiomyoblasts. Concentrations of 1, 3, and 5 µM were selected for elucidating the effect of butein on oxidative-stress-inducible cells.

### 3.2. Butein Protects H9c2 Cardiomyoblasts from H_2_O_2_-Induced Oxidative Stress

**Figure 2:**
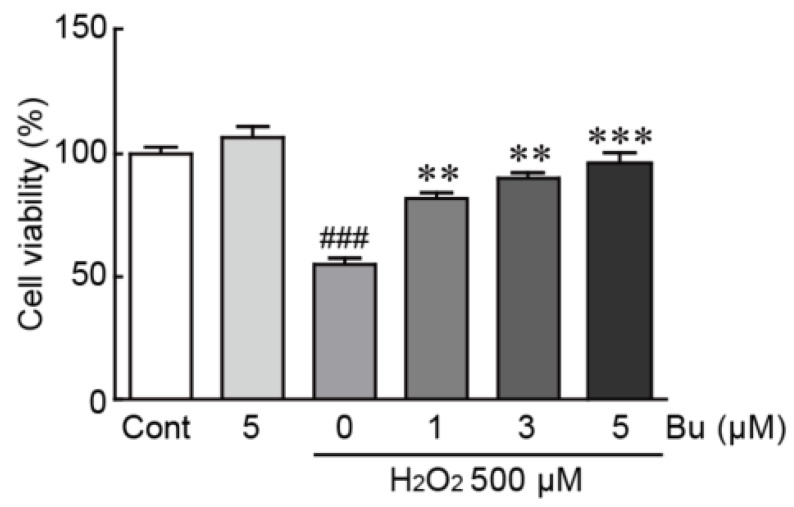
Butein increases the cell viability of H_2_O_2_-treated H9c2 cardiomyoblasts. The cell viability was determined by MTT assay after treating with butein and/or H_2_O_2_. The results are shown as the mean ± SEM in triplicate. ### *p* < 0.001 vs. control group. ** *p* < 0.01 and *** *p* < 0.001 vs. H_2_O_2_-alone group. Cont; control, Bu; butein.

To induce oxidative stress in H9c2 cardiomyoblasts, we used H_2_O_2_, which is commonly used to evoke cellular oxidative damage [22]. H9c2 cardiomyoblasts were treated with butein and/or H_2_O_2_ to elucidate the protective effects of butein and were applied to the MTT assay. The cell viability of cells treated with 500 µM H_2_O_2_ alone was significantly reduced, with a 55.2% survival rate, compared with that of control cells (Figure 2). Otherwise, when pretreated with butein, the viability of H_2_O_2_-treated cells increased (78.1%, 88.6%, and 98.8% survival rates in cells pretreated with 1, 3, and 5 µM butein, respectively, compared with those in control cells) (Figure 2). These results indicates that butein prevents H_2_O_2_-induced oxidative stress in H9c2 cardiomyoblasts.

### 3.3. Butein Ameliorates Oxidative-Stress-Induced Apoptotic Cell Death in H9c2 Cardiomyoblasts

**Figure 3:**
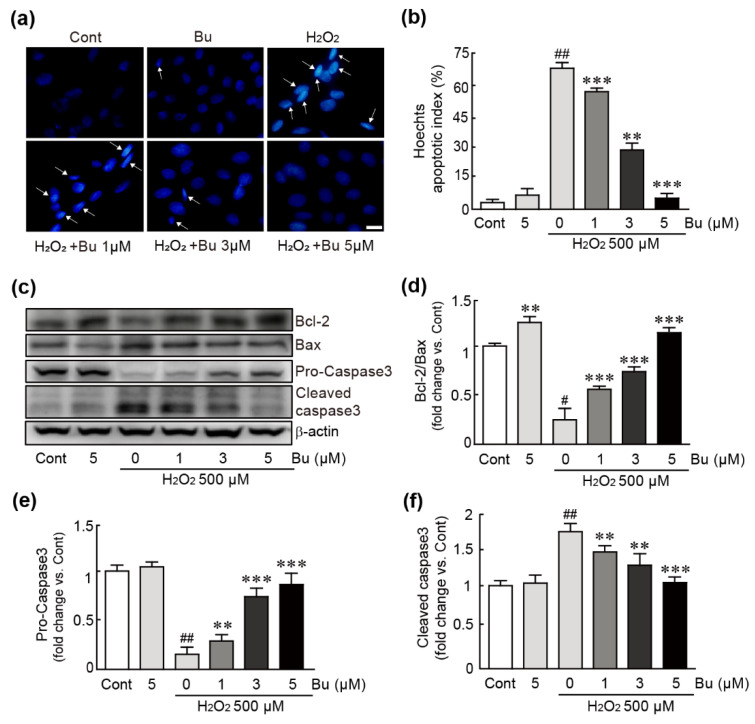
Butein attenuates apoptotic cell death in H_2_O_2_-treated H9c2 cardiomyoblasts. The cells were treated with butein and/or H_2_O_2_ (**a**) Nuclear staining was performed by Hoechst staining. The arrow indicates apoptotic cells with nuclear condensation using cells that were treated with butein and/or H_2_O_2_. The cells in at least 10 fields (30 cells per field) were counted in each group. (**b**) The percentage of apoptotic cells versus total cells is shown as the apoptotic index. (**c**) Western blot analysis for the expression levels of apoptosis-related proteins including Bcl-2, Bax, pro- and cleaved caspase 3, and β-actin (loading control). The band densities of the ratio of Bcl-2/Bax (**d**) and pro- (**e**) and cleaved (**f**) caspase 3 proteins were calculated with HD imaging software. The results are shown as the mean ± SEM in triplicate. # *p* < 0.05 and ## *p* < 0.01 vs. control group. ** *p* < 0.01 and *** *p* < 0.001 vs. H_2_O_2_-only group. Cont; control, Bu; butein. Scale bar, 100 μm.

To evaluate whether butein can suppress apoptosis in H_2_O_2_-treated cells, nuclear staining and Western blotting against apoptosis-related proteins were performed using cells treated with 500 µM H_2_O_2_ or cells pretreated with 1, 3, and 5 µM butein followed by exposure to 500 µM H_2_O_2_. Nuclear staining was performed using Hoechst 33342 dye to detect nuclear condensation as a main feature of apoptosis. The apoptotic cells were significantly increased in H_2_O_2_-only-treated cells (66.8% apoptotic cells/total cells) (Figure 3a,b). Conversely, butein-pretreated cells showed significantly decreased numbers of apoptotic cells in a dose-dependent manner (57.8%, 28.6%, 4.4% apoptotic cells/total cells in 1, 3, and 5 µM butein-pretreated cells, respectively) (Figure 3a,b). The results of Western blotting showed that Bax (pro-apoptotic protein) was significantly increased; the expression level of Bcl2 (anti-apoptotic protein) was decreased in H_2_O_2_-treated cells (0.2-fold change in Bcl-2/Bax in H_2_O_2_-only-treated cells vs. controls). Notably, these changes in expression levels were significantly attenuated by pretreatment with butein (Figure 3c,d). With regard to caspase 3 protein expression as an apoptosis-inducible protein, H_2_O_2_ treatment activated caspase 3 protein: pro-caspase 3, an inactive form, was decreased, and cleaved-caspase 3, an active form, was increased (0.2- and 1.8-fold changes in pro- and cleaved-caspase 3, respectively, in H_2_O_2_-only-treated cells vs. controls) (Figure 3c,e,f).

### 3.4. Butein Attenuates Oxidative Stress in H9c2 Cardiomyoblasts

**Figure 4:**
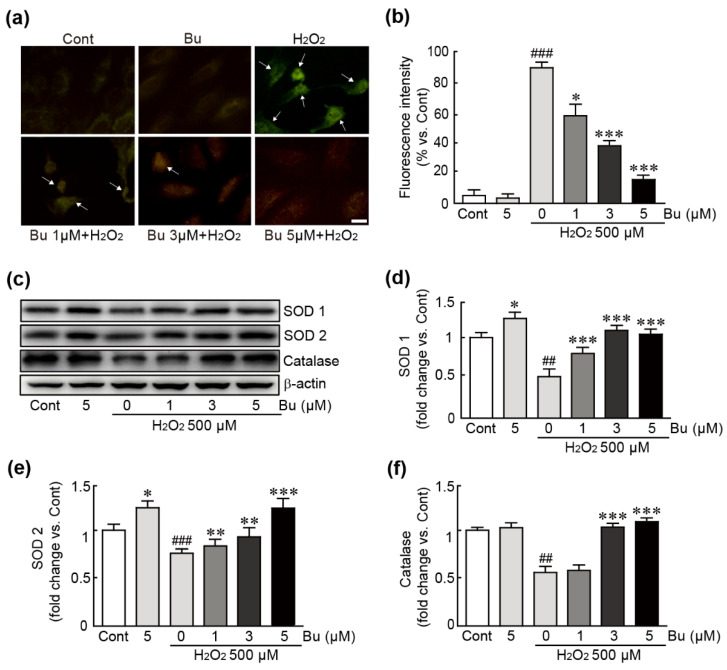
Butein attenuates oxidative stress responses in H_2_O_2_-treated H9c2 cardiomyoblasts. Intracellular ROS production was assessed by a DCFH-DA staining (**a**) and the measurement of green fluorescence intensity (**b**) using cells treated with butein and/or H_2_O_2_. The arrow indicates the cells in which ROS accumulated. (**c**) Western blot analysis for the expression levels of antioxidants including SOD1, SOD2, catalase, and β-actin (loading control). The band densities of SOD1 (**d**), SOD2 (**e**), and catalase (**f**) proteins were calculated with HD imaging software. The results are shown as the mean ± SEM in triplicate. ## *p* < 0.01 and ### *p* < 0.001 vs. control group. * *p* < 0.05, ** *p* < 0.01, and *** *p* < 0.001 vs. H_2_O_2_-only group. Cont; control, Bu; butein. Scale bar, 100 μm.

To determine whether butein inhibits oxidative-stress-induced ROS production in H9c2 cardiomyoblasts, DCFDA dye, an ROS detector, staining was performed in cells treated with butein and/or H_2_O_2_. The results showed that intracellular ROS production detected by green fluorescence was markedly increased in 500 µM H_2_O_2_-only-treated cells. However, pretreatment with butein significantly inhibited ROS production. In particular, 5 µM butein pretreatment reduced ROS production to similar levels in control cells (Figure 4a,b). Western blotting of several antioxidant proteins, including SOD1, SOD2, and catalase, was also performed. The results showed that H_2_O_2_ decreased the expression of these proteins, whereas their expression was significantly increased following pretreatment with butein (Figure 4c–f). Therefore, butein has potential antioxidant capacity by blocking ROS production and restoring the expression of antioxidant proteins.

### 3.5. Butein Attenuates Oxidative-Stress-Induced Mitochondrial Superoxide Production and Dysfunction in H9c2 Cardiomyoblasts

**Figure 5:**
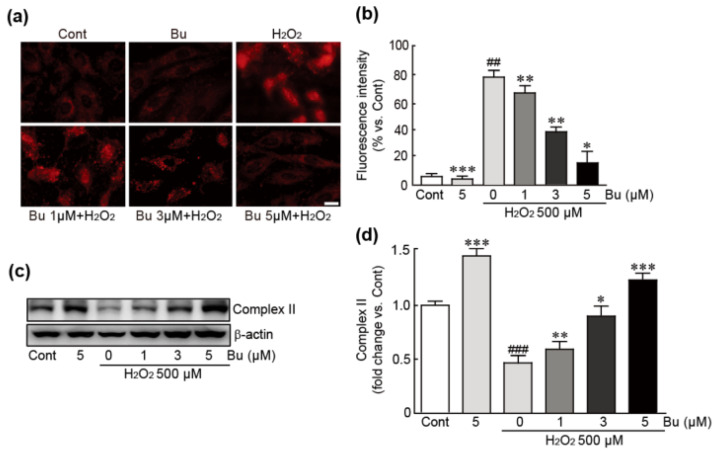
Butein attenuates mitochondrial ROS production and mitochondrial dysfunction in H_2_O_2_-treated H9c2 cardiomyoblasts. Mitochondrial ROS production was measured by MitoSox staining assay (**a**) and red fluorescence intensity (**b**) using cells treated with butein and/or H_2_O_2_. (**c**) Western blot analysis for the expression levels of mitochondrial complex II and β-actin (loading control) in butein-pretreated/H_2_O_2_-treated cells. (**d**) The band densities of the ratio of mitochondrial complex II protein were calculated with HD imaging software. The results are shown as the mean ± SEM in triplicate. ## *p* < 0.01 and ### *p* < 0.001 vs. control group. * *p* < 0.05, ** *p* < 0.01, and *** *p* < 0.001 vs. H_2_O_2_-only group. Cont; control, Bu; butein. Complex II; mitochondrial complex II. Scale bar, 100 μm.

To elucidate whether butein could prevent mitochondrial superoxide production and dysfunction caused by H_2_O_2_-induced oxidative stress, mtSOX Red staining was performed to detect mitochondrial superoxide in cells treated with butein and/or H_2_O_2_. The results showed that H_2_O_2_-only treatment dramatically induced red fluorescence, indicating that mitochondrial superoxide had accumulated (78.7% increase in red fluorescence intensity compared with control cells) (Figure 5a,b). In contrast, lower levels of red fluorescence were observed in butein-pretreated/H_2_O_2_-treated cells (67.1%, 38.6%, and 14.6% increases in 1, 3, and 5 μM butein-pretreated cells, respectively compared with the H_2_O_2_-only treated cells) (Figure 5a,b). Similarly, Western blot analysis against mitochondrial complex II protein as a typical mitochondrial structural protein showed that the expression level of this protein was decreased in H_2_O_2_-only-treated cells; otherwise, butein pretreatment preserved the expression of this protein (0.4-fold change in H_2_O_2_-only-treated cells vs. controls and 0.6-, 0.9-, and 1.2-fold changes in 1, 3, and 5 μM butein-pretreated cells, respectively, vs. H_2_O_2_-only-treated cells) (Figure 5c,d). Collectively, these results demonstrate that butein suppresses oxidative-stress-induced mitochondrial superoxide production and dysfunction caused by H_2_O_2_ in H9c2 cardiomyoblasts.

### 3.6. Butein Inhibits Oxidative-Stress-Induced ER Stress in H9c2 Cardiomyoblasts

**Figure 6:**
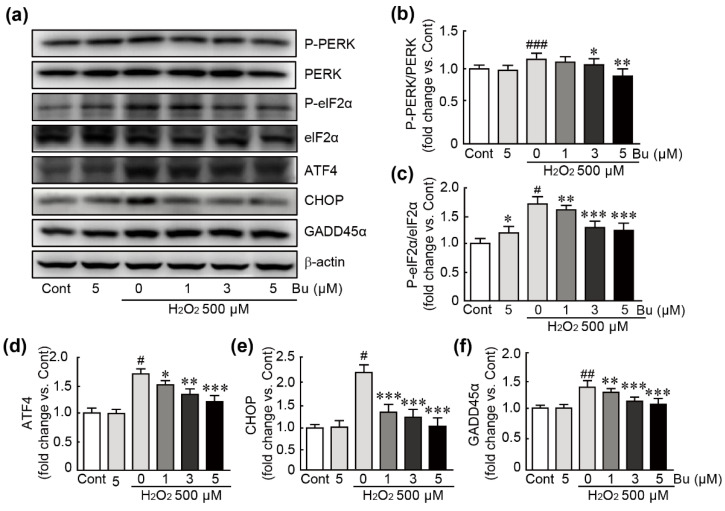
Butein suppresses ER stress in oxidative-stress-induced H9c2 cardiomyoblasts. (**a**) Western blot analysis for the expression levels of ER stress-related proteins including PERK, eIF2α, ATF4, CHOP, GADD45α, and β-actin (loading control) using cells treated with butein and/or H_2_O_2_. The band densities of the ratio of phospho-PERK/total PERK (**b**), ratio of phopho-eIF2α/total eIF2α (**c**), ATF4 (**d**), CHOP (**e**), and GADD45α (**f**) proteins were calculated with HD imaging software. The results are shown as the mean ± SEM in triplicate. # *p* < 0.05, ## *p* < 0.01, and ### *p* < 0.001 vs. control group. * *p* < 0.05, ** *p* < 0.01, and *** *p* < 0.001 vs. H_2_O_2_-only group. Cont; control, Bu; butein.

To evaluate whether butein could affect the ER stress response, which is closely related to oxidative stress, we performed Western blot analysis of proteins involved in ER stress-related signaling pathways. We observed that, after treatment with H_2_O_2_, PERK and eIF2α proteins were activated because the phosphorylation of these proteins was upregulated (Figure 6a–c). In addition, the expression levels of the ATF, CHOP, and GADD45α proteins were significantly increased in H_2_O_2_-only-treated cells (Figure 6a,d–f). Otherwise, butein pretreatment significantly mitigated the upregulation of the expression levels of the aforementioned proteins. In particular, pretreatment with 5 µM butein inhibited the expression levels of these proteins to comparable levels as those in controls, indicating that butein exerts inhibitory effects on oxidative-stress-induced ER stress caused by H_2_O_2_ in H9c2 cardiomyocytes.

### 3.7. Butein Activates the Nrf2-Involved Signaling Pathway in H9c2 Cardiomyoblasts under Oxidative Stress Conditions

**Figure 7:**
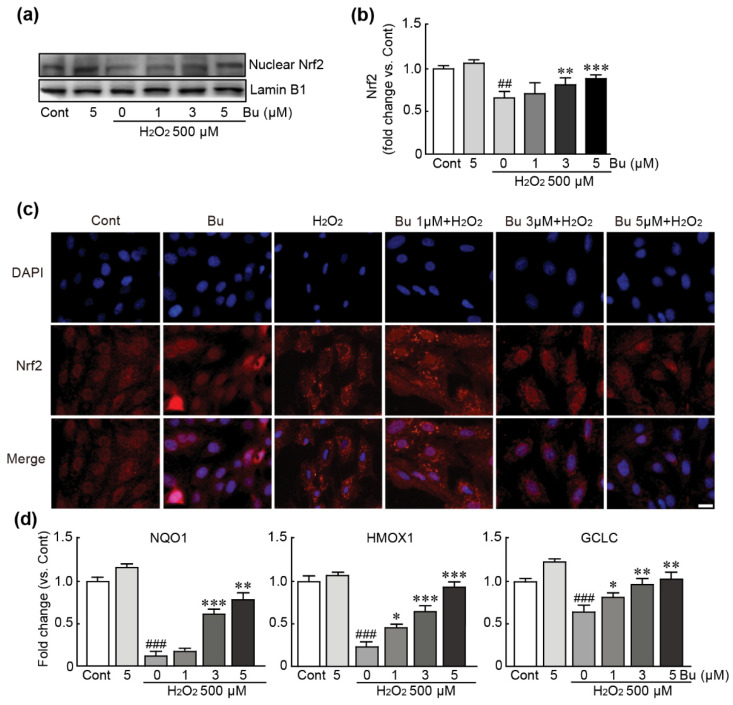
Butein activates the Nrf2-related signaling pathway in oxidative-stress-induced H9c2 cardiomyoblasts. (**a**) Western blot analysis for Nrf2 and lamin B1 (loading control) proteins using the nuclear fraction of cells treated with butein and/or H_2_O_2_. (**b**) The band density of Nrf2 protein was calculated with HD imaging software. The results are shown as the mean ± SEM in triplicate. (**c**) Immunofluorescence images of Nrf2 protein (red fluorescence). The nucleus (blue fluorescence) was stained with DAPI. (**d**) The mRNA expression of Nrf2 target genes including NQO1, HMOX1, and GCLC was performed by qRT-PCR analysis in triplicate. ## *p* < 0.01 and ### *p* < 0.001 vs. control group. * *p* < 0.05, ** *p* < 0.01, and *** *p* < 0.001 vs. H_2_O_2_-only group. Cont; control, Bu; butein. Scale bar, 100 μm.

Nrf2, a transcription factor, has been shown to play a pivotal role in antioxidant responses [23]. Therefore, we determined whether Nrf2 signaling is involved in the antioxidant activities of butein in H9c2 cardiomyoblasts with oxidative stress induced by H_2_O_2_. Nrf2 protein in nucleus was decreased in H_2_O_2_-only-treated cells; otherwise, the nuclear-expression levels of Nrf2 were markedly increased in a dose-dependent manner when butein pretreatment was applied to H_2_O_2_-exposed cells. In particular, pretreatment with 5 µM butein significantly increased the expression level of this protein compared with that in control cells (Figure 7a,b). Similarly, immunostaining for the Nrf2 protein also showed that the Nrf2 protein was translocated from the cytoplasm to the nucleus when butein was used to pretreat H_2_O_2_-treated cells. (Figure 7c). Finally, the qRT-PCR analysis of several Nrf2 target genes with antioxidant properties, including NQO1, HMOX1, and GCLC, was performed. In H_2_O_2_-only-treated cells, the expression levels of these genes were markedly decreased compared with those in control cells (0.1-, 0.2-, and 0.7-fold change in NQO1, HMOX1, and GCLC in H_2_O_2_-only-treated cells, respectively, compared with controls) (Figure 7d). However, the expression levels of these genes were significantly increased when 3 µM or 5 µM butein was used to pretreat in H_2_O_2_-exposed cells (Figure 7d). Collectively, these results indicate that butein activates the Nrf2-related signaling pathway in H9c2 cardiomyoblasts with oxidative stress induced by H_2_O_2_.

## 4. Discussion

In recent years, antioxidant therapy has been attracting attention to improve heart function to protect against a variety of cardiovascular diseases, such as heart failure, myocardial infarction, and ischemic heart diseases [24]. As part of these efforts, many antioxidants have been discovered and contribute to maintain heart function to protect against the heart diseases mentioned above [25]. Since it was identified from the tuber of *Hydnophytum formicarum Jack* in 2008, butein has been reported to possess antioxidant properties [26]. Many previous studies have been reported that butein is involved in the development of a variety of chronic diseases, such as cancers [27,28], nephritis [29], obesity [30], diabetes [31], and hypertension [32]. In particular, a recent study demonstrated that butein protected heart function from oxidative injury through the extracellular signal-regulated kinase (ERK)/Nrf2 signaling pathway in a chronic heart failure rat model [33]. However, an in-depth study focusing on oxidative stress itself has not been conducted. Therefore, the protective effect of butein against oxidative stress in the heart and its underlying mechanisms need to be elucidated. In the present study, we observed the potential antioxidative properties of butein in H9c2 cardiomyoblasts with oxidative stress induced by H_2_O_2_.

First, we demonstrated that butein markedly increased the viability of cardiac cells after treatment with H_2_O_2_, which is an oxidative stress inducer. Since previous findings indicated that oxidative stress causes cell apoptosis [34], we then tested whether butein could block apoptotic cell death after inducing oxidative stress in cardiac cells. The assays related to apoptotic cell death and Western blot analysis for proteins in the apoptosis signaling pathway demonstrated that butein significantly inhibited DNA damage, which is a main feature of apoptosis, and the expression of apoptosis-inducing proteins in cardiac cells under oxidative stress. Therefore, these results demonstrated that butein effectively rescued cardiac cells from oxidative stress.

We next elucidated the anti-oxidative effects of butein in cardiac cells with oxidative stress induced by H_2_O_2_. Although H_2_O_2_ has a critical role in the normal function of cells through acting on various cell signaling pathways [35], it also simultaneously causes oxidative damage [36]. Herein, the effects of butein on ROS production and the expression levels of antioxidants were measured. A DCFH-DA assay showed that butein dramatically decreased intracellular ROS production in cardiac cells after treatment with H_2_O_2_. Consistent with the suppression of ROS production, treatment with butein also attenuated the decreased levels of the typical antioxidants, including catalase, SOD1, and SOD2 by exposure to H_2_O_2_. Thus, these results suggest that butein effectively blocks oxidative stress caused by H_2_O_2_ in H9c2 cardiomyoblasts.

Increasing evidence has suggested that mitochondrial dysfunction is closely associated with the incidence of oxidative stress [10]. Indeed, the mitochondria are prominent sources of ROS production. More specifically, the mitochondrial respiratory chain in mitochondria plays an important role in energy production in which electrons move across the inner mitochondrial membrane to synthesize ATP [37]. The electrons interact with oxygen and further produce superoxide or H_2_O_2_, contributing to oxidative stress. Under normal conditions, mitochondria utilize an exquisite network of ROS scavenging systems to eliminate mitochondrial ROS including SODs, catalase, the GSH-PX system, and the PRX/Trx system [38]. However, mitochondrial abnormalities in the heart in pathological states cannot properly operate these ROS scavenging systems and cause the further dysregulation of mitochondrial ROS [39]. In accordance with this, our results showed that H_2_O_2_ treatment caused mitochondrial dysfunction and increased mitochondrial ROS production. Notably, pretreatment with butein significantly attenuated mitochondrial dysfunction and ROS production.

The ER is a specialized subcellular organelle that is essential for protein folding, secretion, and post-translational modifications [40]. This process within the ER lumen is carefully regulated to maintain the production of normal proteins through the ER quality control system [41]. However, when this control system is perturbed due to numerous physiological or pathological stimuli, the accumulation of unfolded and misfolded proteins is triggered, which is known as ER stress [42]. ER stress consequently promotes cellular damage including apoptosis and oxidative stress. In particular, in the ER under stress conditions, dysregulated S-S bond formation and the cleavage of proteins result in ROS production and further cause oxidative stress [43]. Recent studies have shown that ER stress is closely associated with the incidence of a variety of cardiovascular diseases, including cardiac hypertrophy, heart failure, ischemic cardiac diseases, and cardiac fibrosis [40,44,45]. Therefore, we determined the preventive effect of butein on ER stress in H_2_O_2_-treated H9c2 cardiomyoblasts. Herein, we detected that H_2_O_2_-treated cardiac cells showed the activation of or increased expression levels of several ER stress-related proteins. Notably, these changes were dramatically blocked by pretreatment with butein. Collectively, our results demonstrated that butein effectively protected H9c2 cardiomyoblasts from oxidative stress through the inhibition of myocardial dysfunction and ER stress.

To elucidate the mechanism of the effects of butein against oxidative-stress-inducible H9c2 cells, we focused on the Nrf2-related signaling pathway. Nrf2, which is a transcriptional factor, has beneficial actions, such as antioxidant and anti-inflammatory responses, against numerous pathological conditions by upregulating the expression of a variety of cytoprotective genes [46]. In particular, this protein induces many genes encoding antioxidant enzymes, such as NQO1, SODs, HMOX1, GCLC, and GCLM and consequently drives the protective responses against oxidative stress [47]. Accumulating evidence suggests that Nrf2 protein is a critical positive regulator for maintaining heart function in many cardiac diseases [48,49]. In particular, in a previous study using both Nrf2-knockout mice and overexpressing cardiac cells, severe heart failure and increased mortality were noted after the development of cardiac hypertrophy in Nrf2-knockout mice; on the other hand, the overexpression of Nrf2 led to the inhibition of ROS production and increased cardiac fibrosis [50]. Consistent with these reports, our results showed that butein translocated Nrf2 protein from the cytosol to the nucleus, further activating various Nrf2-target antioxidant genes. Therefore, the present study demonstrated that butein prevents the oxidative damage in cardiac cells through the activation of the Nrf2-related signaling pathway.

## 5. Conclusions

The present study demonstrated that butein rescues cardiac cells from the oxidative damage induced by H_2_O_2_. This antioxidant effects of butein are mediated by the preservation of mitochondrial function and inhibition of the ER stress through the activation of the Nrf2 signaling pathway. Therefore, we propose that butein might contribute to the development of a therapeutic strategy for the treatment of oxidative stress in heart diseases.

## Data Availability

Data is contained within the article.

## Supplementary Materials

The following supporting information can be downloaded at: https://www.mdpi.com/article/10.3390/antiox11081430/s1, Table S1: List of antibodies; Table S2: Specific primer sequences for qRT-PCR.
