# Supplementary material for: Butein Ameliorates Oxidative Stress in H9c2 Cardiomyoblasts through Activation of the NRF2 Signaling Pathway
Source: Antioxidants (Basel). 2022 Jul 23;11(8):1430. doi: 10.3390/antiox11081430 (PMC9331242; doi:10.3390/antiox11081430)
Supplement: Supplementary file 1 [file antioxidants-11-01430-s001.zip › antioxidants-1834894-supplementary.pdf]

Supplementary Table S1. List of antibodies

Antibody	Catalog number	Working dilution	Company
B-cell lymphoma 2 (Bcl-2)	sc-492	1:1,000	Santa Cruz Biotechnology
Bcl-2-associated X protein (Bax)	#2772	1:1,000	Cell signaling
pro-caspase 3	#9665	1:1,000	Cell Signaling
superoxide dismutase 1 (SOD1)	sc-101523	1:1,000	Santa Cruz Biotechnology
superoxide dismutase 2 (SOD2)	#1341	1:1,000	Cell signaling
catalase	#14097	1:1,000	Cell signaling
mitochondrial complex II protein	MS204	1:1,000	Abcam
pancreatic ER kinase (PERK)	#3192	1:1,000	Cell signaling
phosphorylated PERK (p-PERK)	ab192591	1:1,000	Abcam
eukaryotic translation initiation factor 2α (eIF2α)	#9722	1:1,000	Cell signaling
phosphorylated eIF2α (p-eIF2α)	#3597	1:1,000	Cell signaling
activating transcription factor 4 (ATF4)	#11815	1:1,000	Cell signaling
C/EBP homologous protein (CHOP)	#2895	1:1,000	Cell signaling
growth arrest and DNA damage-inducible protein 45α (GADD45α)	#4632	1:1,000	Cell signaling
Nrf2	sc-365949	1:1,000	Santa Cruz Biotechnology
β-actin	sc-47778	1:1,000	Santa Cruz Biotechnology
Lamin B1	Sc-374015	1:1,000	Santa Cruz Biotechnology

Supplementary Table S2. Specific primer sequences for qRT-PCR

Genes	Accession No.	Primers	
NQO1	NM_017000	forward	5′-TGAGCCCGGATATTGTAGCTGA-3′
		reverse	5′-GCATACGTGTAGGCGAATCCTG-3′
HMOX1	NM_012580	forward	5′-ATTTGTCCGAGGCCTTGAA-3′
		reverse	5′-CCAGGGCCGTATAGATATGGTA-3′
GCLC	NM_012815.2	forward	5′-GTGGACACCCGATGCAGTA-3′
		reverse	5′-CTTGTAGTCAGGATGGTTTGCAATA-3′
GAPDH	NM_017008	forward	5′-CTCTACCCACGGCAAGTTC-3′
		reverse	5′-GCCAGTAGACTCCACGACATA-3′

NQO1; NAD(P)H dehydrogenase, quinone, HMOX1; heme oxygenase 1, GCLC; glutamate-cysteine ligase, catalytic subunit, GAPDH; glyceraldehyde 3-phosphate dehydrogenase
